# Microscopy and elemental analysis characterisation of microplastics in sediment of a freshwater urban river in Scotland, UK

**DOI:** 10.1007/s11356-019-04678-1

**Published:** 2019-03-08

**Authors:** Reina M. Blair, Susan Waldron, Vernon R. Phoenix, Caroline Gauchotte-Lindsay

**Affiliations:** 1 School of Geographical and Earth Sciences, University of Glasgow, Glasgow, Scotland G12 8QQ UK; 2 School of Geographical and Earth Sciences, University of Glasgow, Room 211, Main Building, East Quad, Glasgow, G12 8QQ UK; 3 Department of Civil and Environmental Engineering, University of Strathclyde, Glasgow, Scotland G1 1XQ UK; 4 School of Engineering, University of Glasgow, Glasgow, Scotland G12 8LT UK

**Keywords:** Microplastic, Plastic pollution, Electron microscopy, Fibres, Freshwater, Sediment

## Abstract

Understanding of the sources, fate, and impact of microplastics (MPs, < 5 mm) remains limited, particularly in freshwater environments, while limited comparability across available surveys hinders adequate monitoring and risk assessment of these contaminants. Here, the distribution of microscopic debris in an urban river close to the marine environment in the West of Scotland was investigated to assess concentration and distribution of primary and secondary MPs. Also, the efficiency of light and scanning electron microscopy with energy-dispersive spectroscopy (SEM-EDS) was evaluated for characterisation and quantification of MPs sized 2.8 mm–11 μm. Bank sediment samples were collected twice from the River Kelvin in Glasgow and were size-fractionated and processed for extraction of MPs by density separation. Sample MPs spiking and use of procedural blanks allowed the influence of processing on field data quality to be considered. Total abundances were 161–432 MPs kg^−1^ dry sediment, with fibres as the dominant type, comprising > 88% of total counts. Nevertheless, fibres in blanks suggest potential contributions from atmospheric contamination. Moreover, fibres concentrated mainly in fractions < 0.09 mm suggesting that their fate may be influenced by drivers of fine sediment dynamics in rivers. While no primary MPs were observed, metallic and glass pellets were present in high abundances in settled material and could be easily misidentified by visual inspection, demonstrating that compositional analysis is needed to avoid analytical errors from MP misidentification and overestimation. SEM-EDS allowed for a quick screening of plastic vs non-plastic pellets and improved identification of smaller fragments, whereas more advanced techniques are needed for proper identification of fibres. This study is the first to report on MPs in freshwater rivers in Scotland and suggests that diffuse sources of pollution may be delivering secondary MPs to the river. Their sources, fate, and risk in these systems will thus warrant further attention.

**Electronic supplementary material:**

The online version of this article (10.1007/s11356-019-04678-1) contains supplementary material, which is available to authorized users.

## Introduction

Plastic production and subsequent pollution are global environmental concerns. Global plastic generation has exhibited an upwards trend since the 1950s, reaching 335 million tonnes in 2016, a 10% increase from 2015 levels (Plastics Europe 2017). Moreover, an estimated 8300 million metric tonnes of plastic have been produced since 1950 to date, with approximately 6300 million metric tonnes of plastic waste created until 2015, of which only 9% was recycled (Geyer et al. 2017). Plastics are persistent materials, so when discarded as waste, they can accumulate in landfills and the environment for a long time (Geyer et al. 2017) and pose a threat to biodiversity, ecosystems services and potentially human health (Eerkes-Medrano et al. 2015).

Arising from its aesthetic and environmental impacts, plastic contamination has received increasing attention from the public and scientific communities for several decades (Coe and Rogers 1997; Derraik 2002; Blair et al. 2017), especially larger, visible pieces. Of recent concern is microscopic plastic debris commonly referred to as microplastics (MPs), typically less than 5 mm in size (GESAMP 2015), although a formal definition and lower limit have not been established (Blair et al. 2017). They are divided, broadly, into primary or secondary types (GESAMP 2015), though these definitions are also not standardised. Primary MPs are produced intentionally and are typically small spherical pellets that can originate from their use in cosmetic and personal care products, as sand-blasting media, and pre-production pellets commonly known as “nurdles” (Storck and Kools 2015). Secondary MPs, such as fibres, fragments, and flakes, are formed indirectly from the breakdown of larger plastic pieces. Sources of secondary MPs may be mismanaged plastic litter, release of fibres through everyday use and washing of synthetic textiles (Browne et al. 2011; Boucher and Friot 2017), and wear and tear of tyres, road markings and paints (Boucher and Friot 2017). Primary MPs have garnered the most media and public attention, prompting actions worldwide sometimes leading to country-wide bans on the use of microbeads (e.g. in the Netherlands, Canada, USA, UK and New Zealand). Despite the greater focus on primary MPs, secondary types may be of increasing abundance, particularly fibres released into wastewater via washing machine effluent (Browne et al. 2011). Fragmented secondary MPs may increase in quantity over time, long after primary inputs are reduced since larger pieces may continue to degrade into smaller plastic particles. Currently, the contribution of different sources to overall MP loadings to the environment and the relative importance of primary and secondary types remains poorly understood (Duis and Coors 2016; GESAMP 2015).

Research focused on understanding the sources, distribution, fate and impact of MP fractions in the environment is increasing rapidly (Blair et al. 2017; Horton et al. 2017a), but knowledge of MP pollution in oceans compared to freshwater environments remains more advanced (Thompson et al. 2009; Wagner et al. 2014; Eerkes-Medrano et al. 2015). Coastal and beach surveys conducted between 1980 and 2001 worldwide revealed that plastic waste can account for 50–90% of all marine litter and that MP materials have been accumulating rapidly in oceans and shorelines over the past few decades (Derraik 2002). More recently, interest in MPs in freshwater systems has been rising (Eerkes-Medrano et al. 2015) as these are known to be important transport vectors of land-based contaminants to coastlines and open sea environments. Widespread MP abundances have been observed in river and lake surveys of water and sediment samples collected from North American, Asian, and European locations (Blair et al. 2017) with the highest concentrations in freshwaters to date observed in highly contaminated areas of Lake Taihu, China (Su et al. 2016), and in sediment of the River Tame (Hurley et al. 2018). Nevertheless, the role of fluvial waters as conduits of MPs to the marine environments from terrestrial sources has been largely unknown due to a lack of empirical data, although this is a rapidly growing field. Investigating the abundance and nature of MPs in rivers close to estuarine and marine environments, particularly in urban and industrialised catchments where MPs could be higher (Nizzetto et al. 2016; Hurley et al. 2018), can potentially further our understanding of this link.

Globally, there is high variability regarding MP abundances and distribution of primary and secondary types (Blair et al. 2017). This may be because MPs are highly diverse in shape, size, colour and density, resulting in high variability in their distribution in space and time, even within localised environmental compartments. Thus, it is important to increase spatio-temporal coverage and generate further local and regional datasets to improve our understanding of this variability. Nevertheless, the diverse nature and small sizes of MPs render them difficult to measure and monitor (Hidalgo-Ruz et al. 2012; Tagg et al. 2015). Consequently, there is a lack of unified research methodology for isolation, identification and quantification of MPs both in oceans and freshwaters, reducing comparability among available surveys. Differences in sampling, density separation and sample digestion techniques, and visual assessment of MPs exist (Hidalgo-Ruz et al. 2012). Recently, analytical techniques have been employed more frequently to determine the chemical composition of the recovered pieces, a step that is important for discriminating MPs from other confounding materials that may be mistaken for plastics, for example cellulose fibres (Wesch et al. 2016). Current methodological limitations can lead to errors in characterisation and quantification of MPs from environmental samples; thus, method validation of extraction and identification protocols should be routinely tested to understand where uncertainty can be introduced and improve the ability to characterise confidently.

This study sought to determine the prevalence and distribution (size, type and colour) of MPs in a site representing of sediment accumulation in the River Kelvin in the west end of Glasgow, Scotland, close to its discharge to the Clyde estuary. Combined physico-chemical characterisation approaches based on light microscopy and electron microscopy with energy-dispersive spectroscopy (SEM-EDS) were used for identification and enumeration of microscopic debris from riverbank sediment. These were required to explore the viability of visual identification of MP and the need to draw on instrumental analysis in routine testing for source verification. This study contributes to generation of spatio-temporal datasets and understanding of what methods are needed for extraction and characterisation of MPs from freshwater environments globally.

## Materials and methods

### Site and sampling

**Fig. 1:**
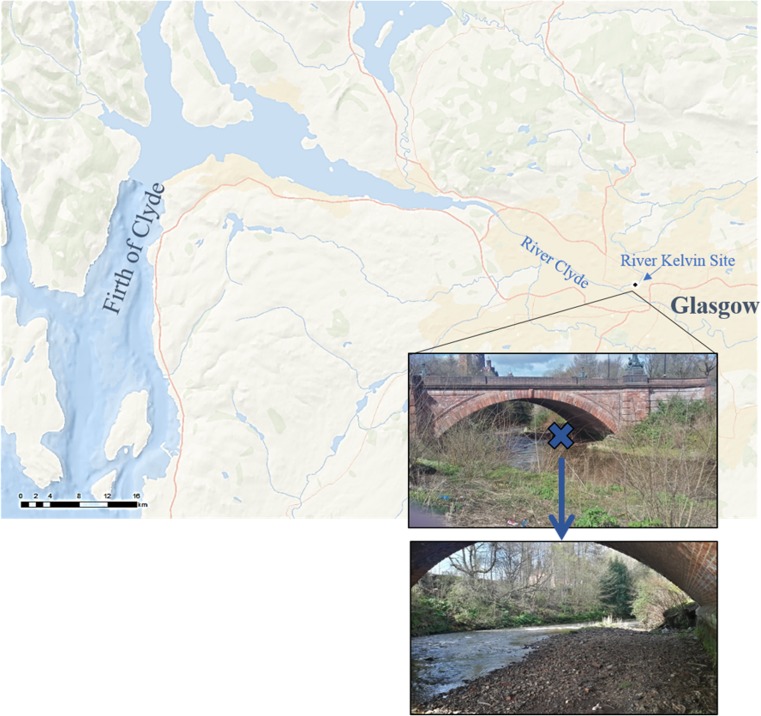
Location of the sample collection site in a river bend section in the River Kelvin in the west of Glasgow, Scotland, UK

The River Kelvin is a freshwater river in Glasgow, UK, rising near Kelvinhead in northern Glasgow and flowing southwest for approximately 34 km through woodland and marshland, and recreational and urban areas (Quadrat Scotland 2002). Near its source, the River Kelvin runs parallel to the Forth and Clyde Canal then gradually increasing in volume, finally converging with the River Clyde Estuary in the west end of Glasgow (Quadrat Scotland 2002). Its close proximity to the marine environment makes it particularly suitable to evaluate the role of fluvial systems in the fate and transport of MPs from continental to oceanic waters. Bulk sediment samples from the surface to a depth of 8 and 10 cm, respectively, were collected with a spade in December 17, 2015 (sampling event 1, SE1), and February 15, 2016 (sampling event 2, SE2), from the River Kelvin bank (55°52′8.742″, − 4°17′19.0278″, Fig. 1). The sample site was selected to be representative of dense urban environments with nearby businesses, tourist attractions and residential areas, a road bridge, and a park. The site is located in a low-energy zone in the inner bend where the channel curves underneath the bridge, rendering it geomorphologically favourable for sediment deposition due to low stream energy and reduced velocity. Samples were collected in aluminium tins and wrapped in aluminium foil to avoid contamination by use of plastic containers, and transported to the laboratory 5 min away.

### Sample processing

The methodological approach employed for sample processing broadly follows methods discussed in the literature (Hidalgo-Ruz et al. 2012; Blair et al. 2017). Throughout the process, a white lab coat (65% polyester, 35% cotton) and rubber gloves were used and care was taken to minimise sample contamination by avoiding the use of plastic materials where possible. As the laboratory is a busy environment and it is difficult to control contamination from nearby activities, blanks were used to account for background contamination.

First, samples were weighed in aluminium trays before and after oven-drying for at least 24 h at 100 °C, and mass of total solids (TS) in grams (g) was calculated as the weight of the dried samples. This temperature was selected as the average of methods proposed by Masura et al. (2015) and for standard determination of gravimetric soil moisture (Black 1965); and, as the threshold temperature for melting and decomposition of common thermoplastics (Klein 2011). Using an automatic shaker for a duration of 10 min, oven-dried samples were sieved into the following size classes: 2.8 mm, 2.0 mm, 1.4 mm, 1.0 mm, 0.71 mm, 0.5 mm, 0.355 mm, 0.25 mm, 0.18 mm, 0.125 mm, 0.09 mm, and 0.063 mm, producing 13 sub-samples for each sampling event. Size fractionation was employed to assess how different types of MPs are associated with different sediment grain sizes. Each size class fraction was weighed and stored in a glass bottle until further processing.

### Extraction by density separation

After fractionation, density separation (DS) with a saturated NaCl solution (*ρ* = ~ 1.2 g cm^−3^) was used to separate low-density MP pieces. Approximately 25 g (or entire volume if less than 25 g) of oven-dry sediment from each size fraction was mixed with 40–68 mL of salt solution to cover the sediment, manually shaken vigorously for 1 min and left to settle overnight (~ 24 h). After 24 h, the supernatant was filtered through Whatman 11-μm cellulose filters to collect suspended debris. The filter paper was rinsed three times with deionised (DI) water to remove excess salt and then transferred to petri dishes to dry at room temperature (18–21 °C). During processing of SE1 samples, re-suspension of some settled sediment (i.e. those deposited after the 24 h period) was observed during decanting. Thus, a second settling step was introduced for processing of SE2 samples in which the supernatant was transferred into a clean beaker before filtration, covered, and left to settle for two additional hours to allow for further settling of re-suspended solids and reduce their potential transfer to filters.

The DS extraction method was validated via recovery tests using river bank sediment collected from the same study site, spiked with different types of MP standards. Polyethylene (0.71–0.85 mm diameter, *ρ* = 0.96 g cm^−3^), polypropylene (2.45 mm diameter, *ρ* = 0.866 g cm^−3^) and polystyrene (4.4 mm diameter, *ρ* = 1.048 g cm^−3^) microbeads purchased from Cospheric LLC (Santa Barbara, California) were used to mimic primary MPs. Nylon toothbrush bristles and rope fragments, polypropylene cleaning brush bristles, and polyethylene mesh fruit packaging fragments produced in the lab were used to mimic fibrous secondary MPs. Briefly, approximately 20 g of oven-dried sediment was spiked with 10 beads or 15 fibre-like fragments, in triplicates for each polymer type, thoroughly mixed, and processed the same way as field samples (see “Extraction by density separation” and “Identification and quantification”). Recovery efficiencies were calculated as [number of pieces extracted/number of pieces spiked] × 100).

Procedural blanks consisting of NaCl solution were produced with every filtration sequence to account for background contamination.

### Identification and quantification

First, a stereo microscope was used to identify MPs based on physical appearance. Here, samples different from sediment grains (i.e. more rounded, pitted, fibre-like, coloured or transparent) were identified and counted, and pieces in sizes ranging < 2.8 to 0.7 mm were picked out with metal tweezers into glass vials and photographed with a Leica MC120 HD camera connected to a Leica MX7_5_ microscope with magnification between × 10 and × 32, depending on the size of the particle. Pieces smaller than 0.7 mm were not extracted this way, as they were too small to manipulate and could be lost during manual transfer; these fractions were counted and saved on the filter paper until further instrumental analysis. Settled solids were also inspected under light microscopy to detect presence of high-density polymers (*ρ* > 1.2 g cm^−3^).

Representative aliquots of suspected MPs from each category and size fraction were examined using a FEI Quanta 200F scanning electron microscope (SEM) coupled with energy-dispersive spectroscopy (EDS), enabling determination of elemental composition. The aliquot was selected from the SE1 samples and comprised suspended and settled pieces. Briefly, samples were prepared by placing individual pieces > 0.7 mm on double-sided adhesive carbon discs (9-mm diameter), mounted on 9-mm specimen stubs and imaged by SEM-EDS operating at an accelerating voltage of 20 keV in the secondary electron and backscattered mode. Suspended pieces < 0.7 mm that could not be separated manually with tweezers were transferred onto the SEM stub by “pressing” the C adhesive over the filter paper and using a light microscope to verify that the target piece was successfully transferred onto the stub. If it was not possible to transfer a piece after multiple tries, a square of filter paper was cut around it and placed on the stub. The compositional data were used to discriminate plastics from non-polymers since the plastics are carbon-based and other materials are expected to be non-organic. Electron microscopy assessment of the aliquot was used to refine the approach to the visual identification of MPs for the remaining samples under light microscopy.

The sum of pieces counted in all size fractions was used to quantify MP abundance for each sampling event by visual characterisation under light microscopy (stage 1) followed by chemical characterisation by SEM-EDS analysis (stage 2) to compare visual and chemical assignation of MPs. Abundances were calculated as [total number of suspected MPs/mass of TS] and expressed in items per kilogram of dry sediment.

## Results and discussion

### Method validation tests and blanks

**Fig. 2:**
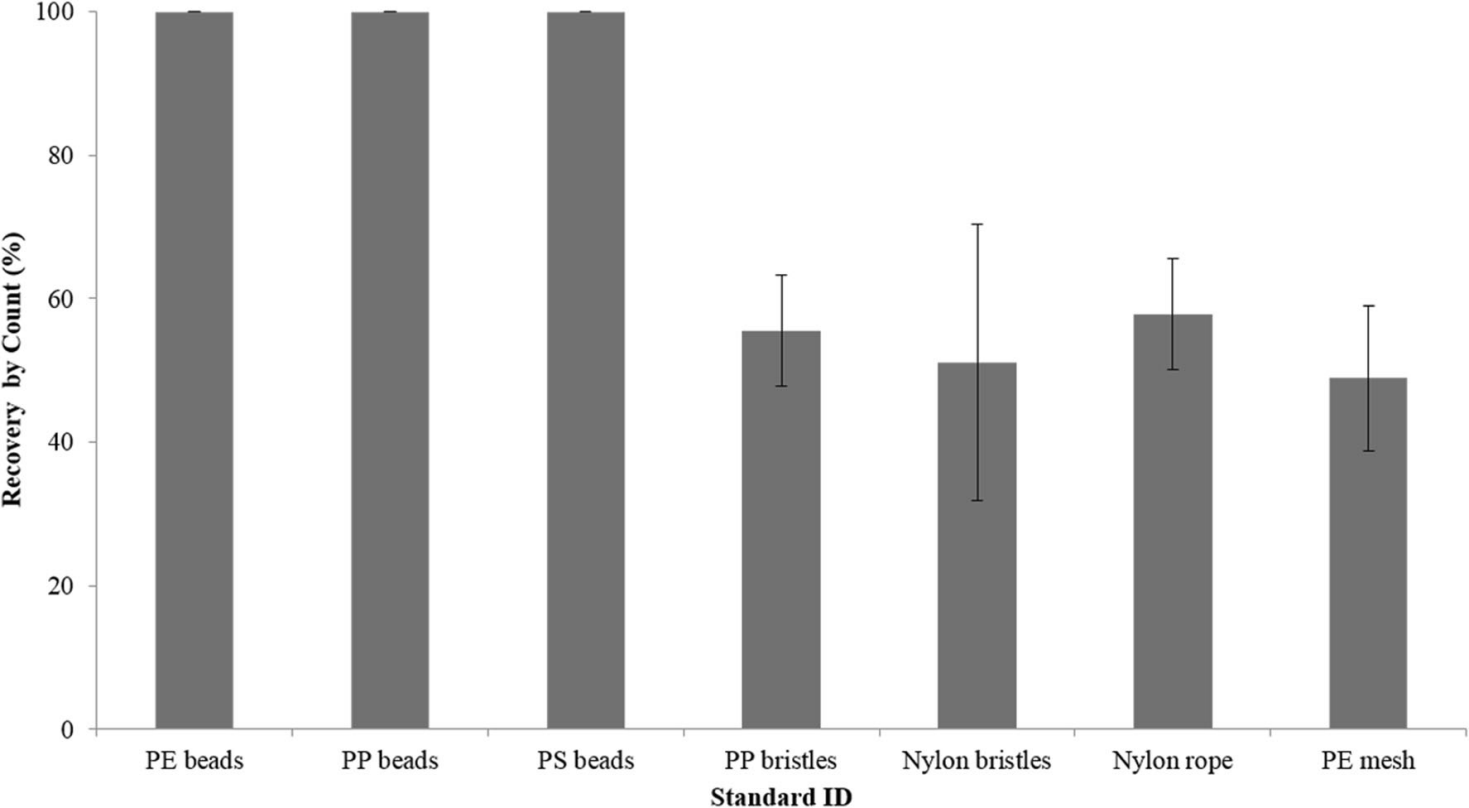
Recovery tests for density separation using various types of microplastic standards: purchased microbeads (polyethylene, PE; polypropylene, PP; and polystyrene, PS), and fibre-like fragments produced in the lab (PP bristles from a cleaning brush, nylon bristles from a toothbrush, nylon rope, and PE mesh packaging)

Recovery rates for MP microbead standards were 100% for all polymer types, sizes, and densities (Fig. 2), while average recovery rates for fibre-like secondary MPs were lower than for primary MPs, ranging from 49 ± 10.2 to 58 ± 7.7% for mesh packaging fragments and nylon rope pieces, respectively (Fig. 2). Lower recovery rates for fibrous MPs may be attributed to a tendency to cluster together and adhere to the inorganic matrix and walls of the container, and may present a challenge for separation and thus accurate quantification of this type of MP.

Fibres were the only type of materials observed in procedural blanks (Table 1). Fibre content in blanks was similar to those observed in other studies (Dris et al. 2015; Horton et al. 2017b; Hurley et al. 2018). Only a handful of freshwater studies have included use of blanks as verification, but when reported, they were considered negligible compared to those observed in field samples (Dris et al. 2015; Horton et al. 2017a) or determined to be non-plastic (Hurley et al. 2018). Thus, the field data were not blank corrected in this study. Nevertheless, their occurrence in blank controls suggests background contamination, meaning that the field samples may contain a non-river contribution of fibres that could result in overestimation. Conversely, their lower recovery rates could result in an underestimation in both the sample and the blank. As fibres seem to be a predominant MP category in this and many studies, more blank and standard control tests are needed to reduce these uncertainties and improve confidence in results.

**Table 1:** Microplastic counts in River Kelvin sediment sampled December 17, 2015 (SE1), and February 15, 2016 (SE2), by category, and total counts and abundance aggregated across all size fractions for stages 1 (visual characterisation) and 2 (chemical characterisation)

Identification stage	Sampling event	Sediment weight, dry (g)	Microplastics count (*n*)					Abundance (items per kg dry sediment)
			Pellets	Fibres	Fragments	Other	Total	
Visual (stage 1)	SE1	441.49	5	64	23	5	97	220
	SE1 blanks (*n* = 2)	0	0	3	0	0	3	
	SE2	254.48	0	106	8	0	114	448
	SE2 blanks (*n* = 4)	0	0	3	0	0	3	
Chemical (stage 2)	SE1	441.49	0	64	7	0	71	161
	SE2	254.48	0	106	4	0	110	432

### Microplastic categories

**Fig. 3:**
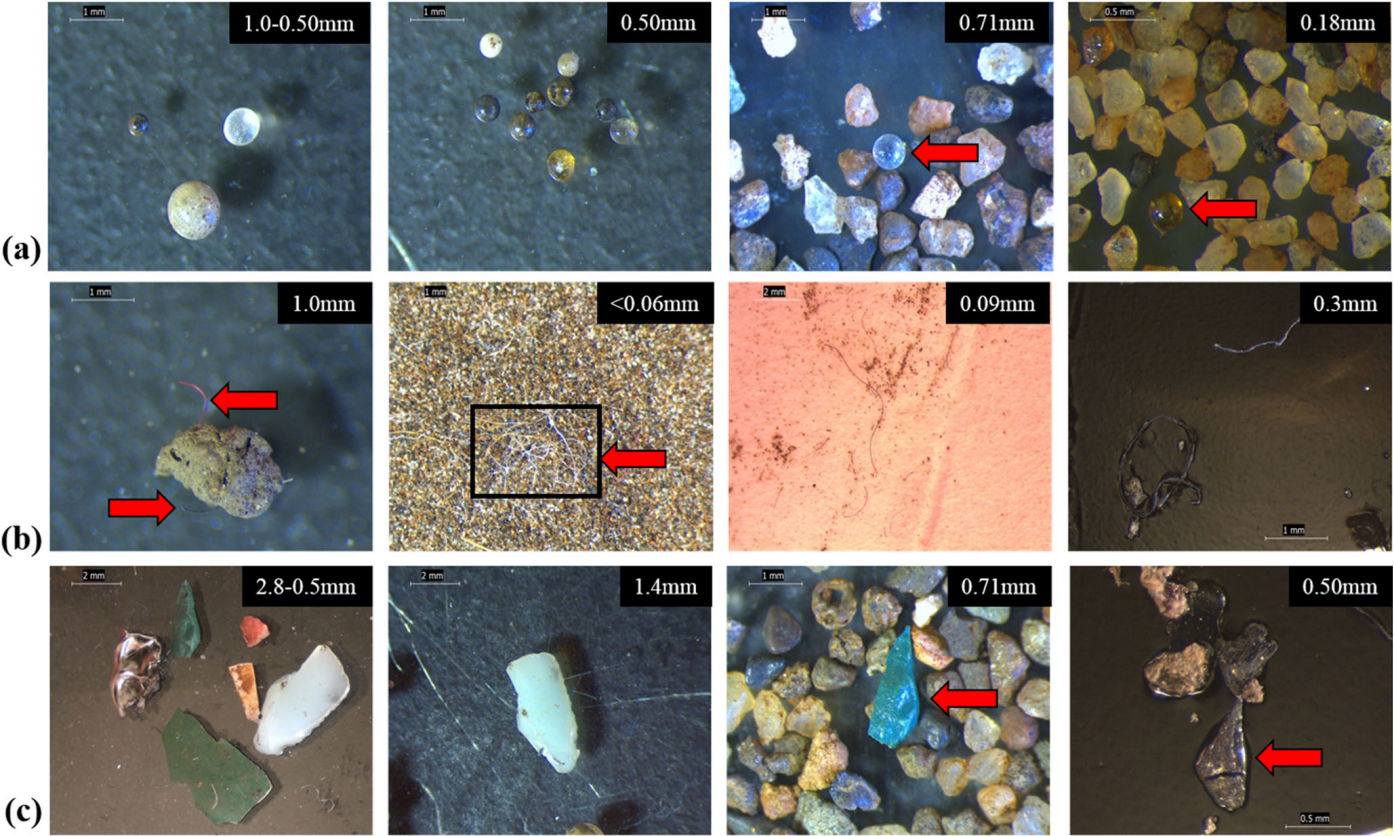
Light microscopy images of suspected microplastics in size-fractionated sediment samples from the River Kelvin in suspended and settled material before chemical characterisation. Items shown are pellets (**a**), fibres (**b**), and fragments (**c**)

Suspected MPs were observed in all size fractions and were classified into three broad categories: (1) pellets, (2) fibres, and (3) fragments (Fig. 3).

#### Micropellets

**Fig. 4:**
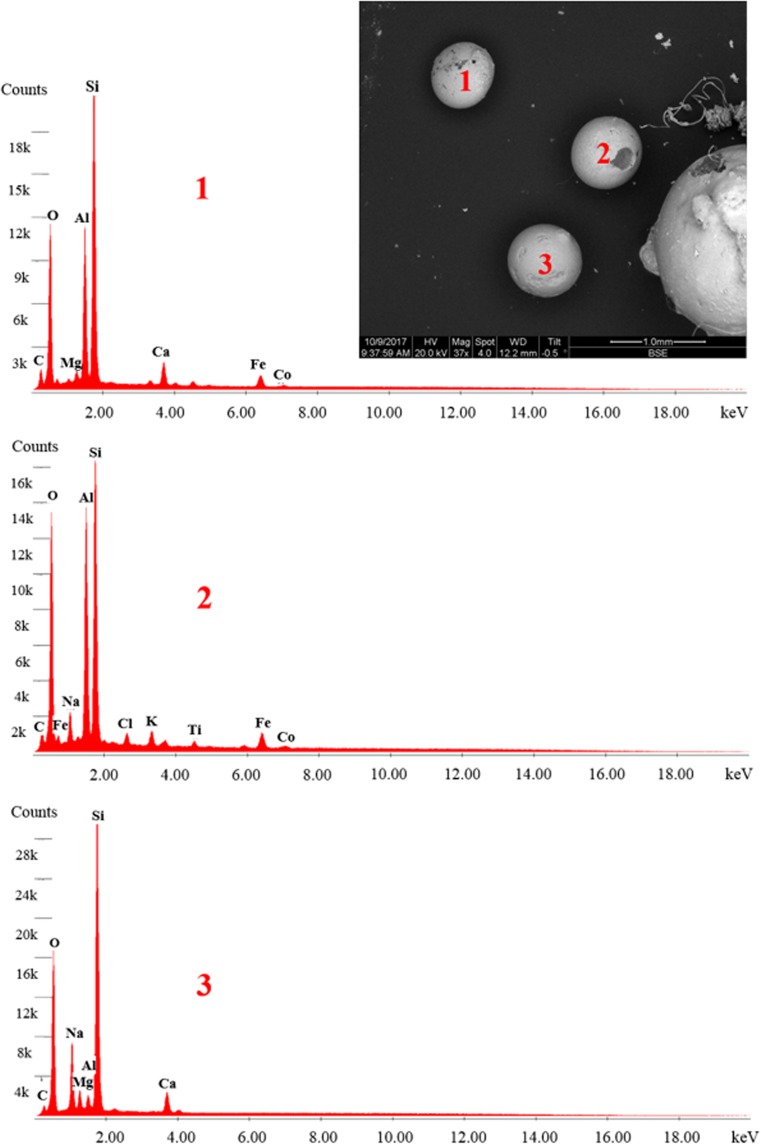
Backscattered electron image and elemental spectra for common micropellets observed in River Kelvin sediment. Pellets were determined to be non-plastic based on absence of a strong carbon signal

At stage 1, five micropellets were observed in suspended material in SE1 only (Table 1), but these were determined to be non-plastic at SE2. Visually, these pellets were dark-coloured and similar in appearance to those reported in a previous study in the St. Lawrence River (Castañeda et al. 2014). Pellets in the St. Lawrence River were determined to be polyethylene microbeads based on chemical characterisation by differential scanning calorimetry; thus, suspended pellets in the River Kelvin were suspected to be also MPs. However, SEM-EDS analysis performed here showed that suspended pellets were primarily metallic (Fig. 4). The physical similarities but differing elemental compositions between the two studies indicate that non-MP pellets can be easily mistaken for MPs by visual inspection alone. The absence of primary MPs in this study contrasts with reports from earlier freshwater studies in urban catchments that found primary MPs to be more common than secondary forms based on visual and chemical characterisation (Zbyszewski and Corcoran 2011; Eriksen et al. 2013; Castañeda et al. 2014; Hurley et al. 2018; Peng et al. 2018). The high recovery rates for pellets from the validation tests provided confidence that, although no MP pellets were isolated from the environmental samples for this study, this was likely due to their absence from the site and not due to extraction error.

Visual examination revealed that micropellets were the predominant type of MPs in settled material by count across all size fractions for December and February samples, respectively (Electronic Supplementary Material). Settled micropellets consisted mostly of dark spheres similar to suspended ones, with a few clear and white- or cream-coloured pieces (Fig. 3a). Micropellets were present mainly in the mid-range particle size fractions (0.25–0.7 mm). These were also present in clusters or aggregations of pellets that appeared to have been fused or melted together. Owing to their physical resemblance to micropellets observed in previous studies (Castañeda et al. 2014), an aliquot of settled pellets representing varying colours and sizes was analysed by SEM-EDS to assess whether they were high-density MPs or non-plastic. The chemical composition was determined to be mostly metallic for dark pieces, while light-coloured pellets were mostly silica (Fig. 4). While these micropellets were not MPs and therefore not the focus of this study, their high concentrations might warrant further evaluation to determine source of origin since they do not occur naturally in the aquatic environments. For example, aluminium silicate pellets could reflect coal fly ash as observed in the Laurentian Great Lakes (Eriksen et al. 2013), while other metallic pellets could be contaminants related to mining and industrial activities similar to those observed in other UK rivers (Rees et al. 1999). If similar in size, shape, and colour as their MP counterparts, these micropellets could also be harmful to the aquatic fauna if ingested. It is also important to be aware of their presence as they could be mistaken for MPs by visual inspection, especially if extracted by density separation as here. As metals have higher density, it would be expected that DS would not extract these materials. In this study, the five pellets in SE1 extracted by DS at stage 1 may be explained by the presence of a porous surface that was only evident during examination of structural composition in SEM-EDS images.

#### Microfibres

Fibres were the most abundant type of suspended microdebris (Table 1), consisting primarily of coloured pieces (i.e. black or dark blue, light blue, and red). Microfibres of similar characteristics were observed in other freshwater ecosystems (Ballent et al. 2016), where fibres < 2 mm identified visually with a stereo microscope were found to be the predominant type of MPs, alongside fragments in the same size range. In the River Kelvin sediment, fibres were observed in isolation, in clusters and embedded in sediment grains (Fig. 3b). Microfibres were observed mostly in the lower size fractions (< 0.090), with the < 0.063-mm size fraction containing nearly 34% and 44% of total fibres in SE1 and SE2 samples, respectively (Electronic Supplementary Material). However, their small sizes and tendency to cluster made it challenging to identify and enumerate visually by light microscopy, especially in the < 0.06-mm fractions (Fig. 3b), potentially leading to their underestimation. No fibres were observed in settled material after DS.

**Fig. 5:**
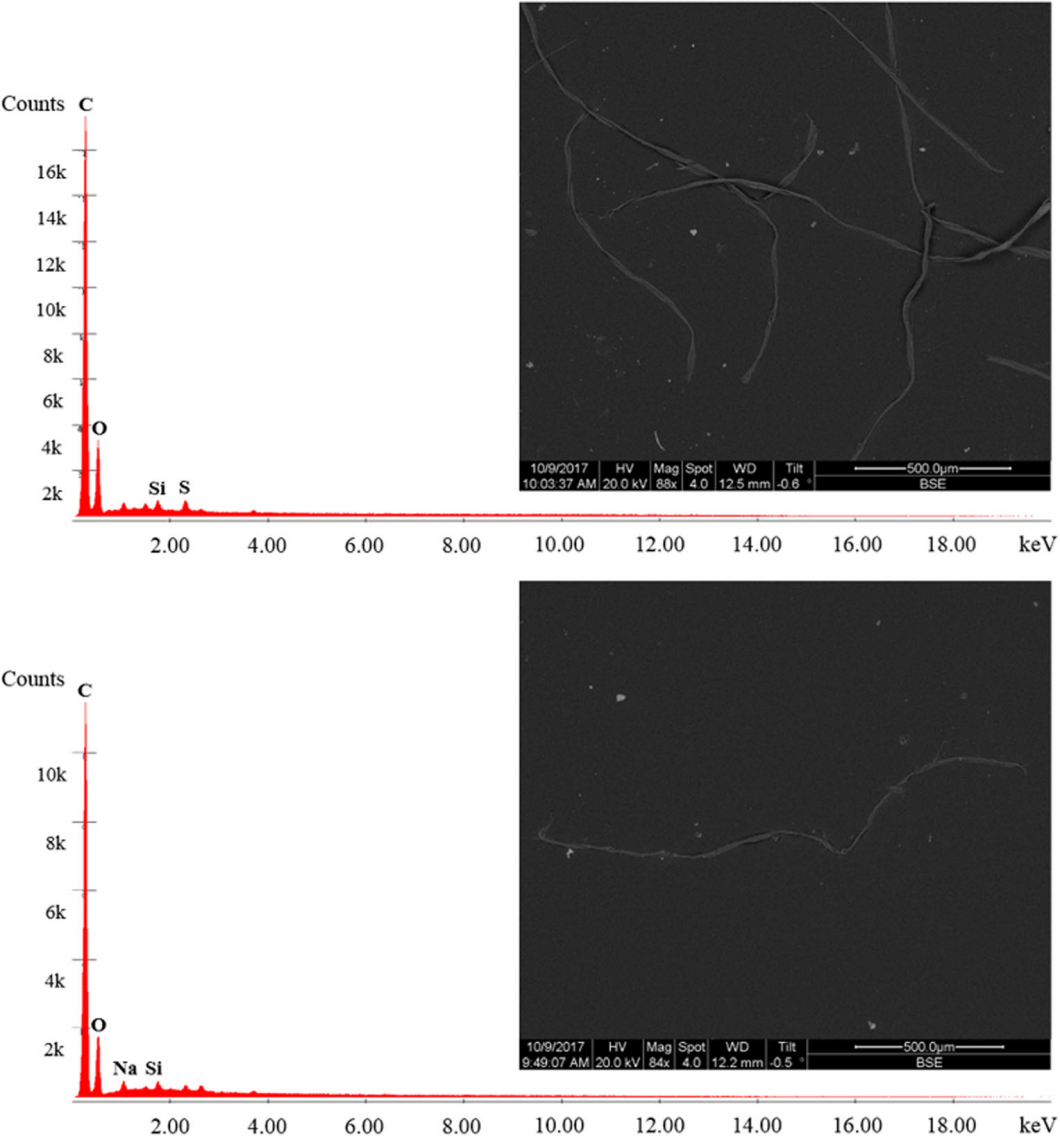
Backscattered electron image and elemental spectra for common microfibres (top) observed in River Kelvin sediment and a 100% cotton fibre standard (bottom). Fibres exhibited a strong carbon signal, but MP could not be discriminated against cellulose fibres

During SEM-EDS analysis at stage 2, fibres exhibited a strong C peak, sometimes accompanied by a smaller O peak (Fig. 5). Therefore, fibres could not be dismissed as non-plastic from their density and chemical composition, resulting in equal counts at stages 1 and 2. Fibres comprised approximately 88% and 95% of all plastic pieces in SE1 and SE2, respectively, in the final enumeration. However, other non-plastic fibres such as cellulose-based ones can exhibit a similar structure and C signal (Remy et al. 2015), and SEM-EDS does not allow for distinction between them (Fig. 5). Spectroscopy analysis via FTIR and Raman has been used successfully for further isolation of MP from non-MP fibres (Remy et al. 2015), highlighting the need for advanced chemical characterisation tools for proper MP quantification, especially in the case of fibres.

Similarly, others have reported the predominance of fibres (Ballent et al. 2016; Su et al. 2016), especially in systems associated with wastewater treatment, as such fibres typically break off synthetic textiles and are released via household sewage (Browne et al. 2011; Magnusson and Norén 2014). While the selected site in the River Kelvin is not located near a discharge pipe from a wastewater treatment facility, it has been suggested that fibres can be transported for greater distances (Ballent et al. 2016); thus, their presence may be attributed to distant inputs upstream from the study site. Conversely, a portion of fibres observed in the samples may be explained by atmospheric fallout of airborne fibres, which can be corroborated by fibre content in rooftop samples collected in urban Paris (Dris et al. 2015) and the presence of microfibres in our procedural blanks (see “Method validation tests and blanks”). While fibre content in blanks could be a result of aerial deposition of fibres released during wear and tear of lab gear, additional deposition of airborne materials into the open channel may occur in the field and account for a portion of fibres observed in river sediment. Furthermore, fibre content in drinking tap water tested in multiple countries (Kosuth et al. 2018) may suggest potential background contamination of fibres even in water purification systems, but this was not tested here and limited studies on MPs in drinking water are currently available.

#### Microfragments

The third category comprises fragmented or flake-like pieces that had uneven edges and appeared to have broken off larger pieces. Suspected MP fragments were observed in suspended and settled material and consisted mainly of coloured pieces (Fig. 3c). Counts varied between sampling events and quantification stage, and although the highest counts were observed in the 0.71 mm size fraction at stage 1, this was not the case for the final counts, and they did not seem to concentrate around a specific size fraction in a discernible pattern. Because high-density polymers can be present in the environment, all settled fragments that physically resembled plastic materials were counted as suspected MP at stage 1 and analysed for chemical composition. Unlike pellets that consistently had little to no C, and fibres that consistently were mostly C, SEM-EDS signals for fragments were more varied and complex.

**Fig. 6:**
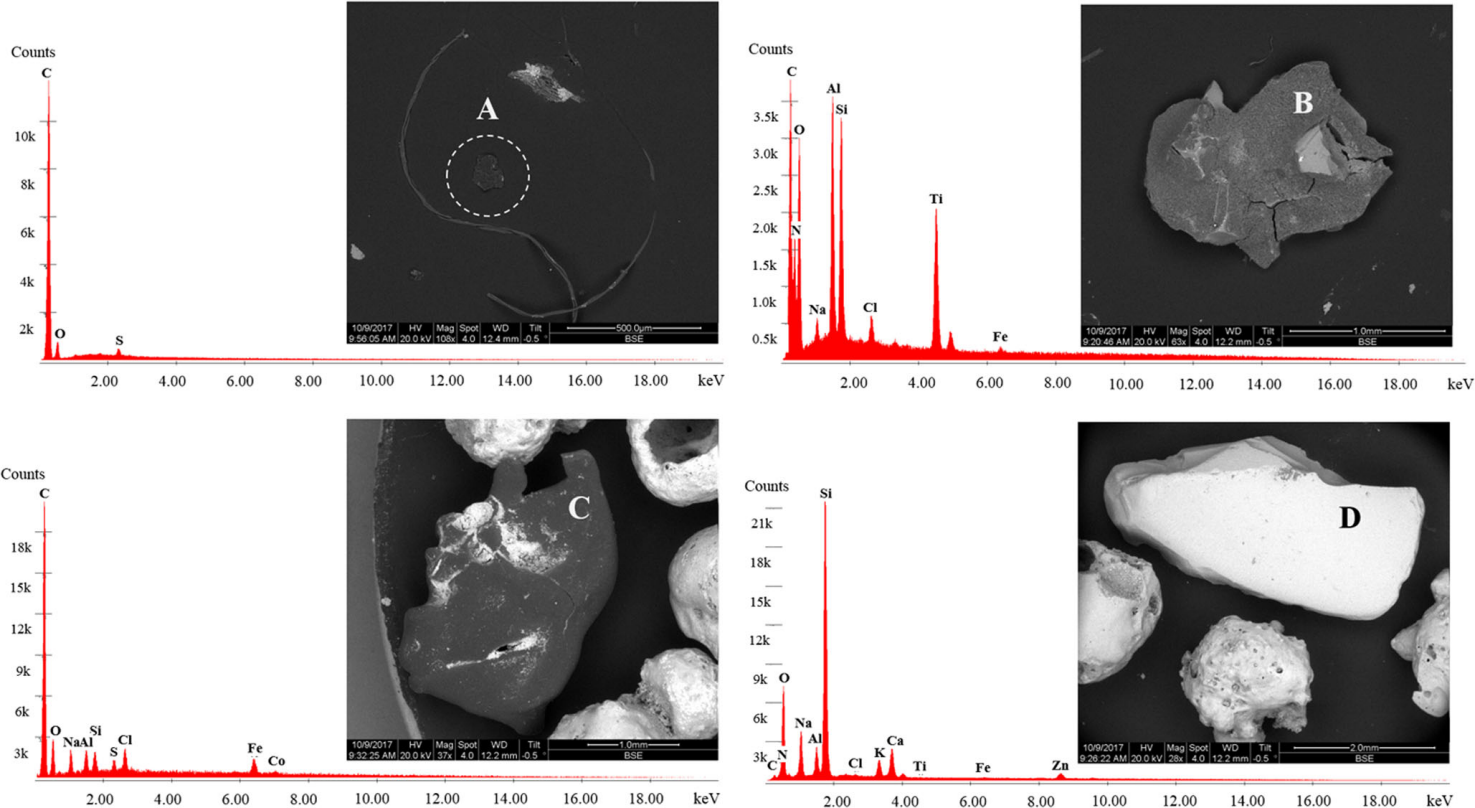
Backscattered electron image and elemental spectra for common microfragments observed in River Kelvin sediment showing floated microplastics (**a**, **b**), settled microplastic (**c**), and settled non-microplastic (**d**) pieces. Pieces were identified as microplastic on the basis of a strong carbon signal

Suspended flake-like fragments with a strong C signal (Fig. 6a) became visible only during SEM-EDS imaging. This is likely explained because these pieces were captured on the filter paper after DS, and, while not visible under light microscopy, they were transferred onto the adhesive while attempting to transfer other materials like fibres using the pressing method. Furthermore, electron microscopy enables greater resolution than light microscopy, making SEM-EDS a powerful tool for detection of smaller pieces like these that may be overlooked by visual inspection, and highlights the detection limits of visual techniques.

Other suspended fragments showed a strong C peak but exhibited additional elemental signals including Ti, Br, and Si (Fig. 6b). These pieces were counted as MPs, due to their strong C signal and low densities, but further analysis via spectroscopy tools (e.g. Raman, FT-IR) should be employed in these cases to identify the type and source of these (and similar pieces) to be conclusive. Only one of ten settled MP fragments showed a strong C signal in the SEM-EDS analysis (Fig. 6c). This may indicate high-density plastic fragments, for example, polyvinyl chloride from construction applications, or polytetrafluoroethylene and engineering polyesters from industrial applications that would need heavier liquids to be extracted (Hidalgo-Ruz et al. 2012). The remaining settled pieces, while initially expected to be plastic due to their bright colours and shapes, showed no carbon signals at stage 2 (Fig. 6d) and therefore were rejected from final counts.

Fragments comprised 12% and 5% of total MP counts in SE1 and SE2, respectively (Table 1). While most studies report either pellets or fibres as the predominant forms of MP debris, and a diversity of fragments generally have been observed across rivers and lakes worldwide, a few studies have reported fragments as the predominant form of these materials in freshwaters systems (Vianello et al. 2013; Wagner et al. 2014; Hurley et al. 2018; Wen et al. 2018; Shruti et al. 2019). Their presence in the catchment may be a result of historical industrial activities or from the fragmentation of plastic litter as the River Kelvin catchment is an area for multiple recreational activities and the sampling site is located underneath a heavily transited bridge near tourist attractions. However, as fragments can originate from the breakdown of larger pieces, their sources may be harder to trace as they are likely to result from non-point pollution, such as rainwater runoff to road drainage systems, losses from landfill sites, riverbanks and floodplains (Kataoka et al. 2019). This is particularly important in MP research as fragments may become more abundant if plastic litter already present in the environment continues to degrade into smaller fractions, and as MPs can further fragment into nanoplastics. Thus, more information on degradation or fragmentation rates of different polymers may play a key role in understanding this category (Hidalgo-Ruz et al. 2012).

### Microplastic abundances

Suspected MP abundance at identification stage 1 supported initial estimates of 220 items per kilogram of dry sediment in SE1 and 448 items per kilogram of dry sediment in SE2. Final MP abundance at stage 2 was 161 and 432 items per kilogram of dry sediment in SE1 and SE2 samples, respectively (Table 1). These concentrations are within ranges observed in other European sites. For example, sediment samples collected from German rivers and inspected visually (Wagner et al. 2014) and chemically (Klein et al. 2015) found 34–64 items per kilogram dry weight in the Rivers Elbe, Mosel, Neckar, and Rhine, and fragments accounted for 60% of total microplastics, with the remainder being fibres (Wagner et al. 2014). However, abundances can be spatially and temporally variable, with other sediment samples from the Rhine yielding 228–3763 items per kilogram, and further 786–1368 items per kilogram in the River Main (Klein et al. 2015). At these sites, the relative abundance of spheres and fragments compared to other shapes was highest in the 63–200-μm and 200–5000-μm size fractions, respectively, while fibres were most abundant in size fractions < 200 μm compared to their concentration in higher size fractions (Klein at el. 2015). In addition, sediment MP abundances in the River Thames were found to range from 18.5 ± 4.2 to 66 ± 7.7 particles per 100 g (equivalent to 185 and 660 particles per kg) of sediment across four sites, with fibres as the main type in three sites and fragments in the fourth, based on visual and chemical characterisation (Horton et al. 2017a). High MP contamination was observed in multiple river channels in the Mersey and Irwell catchments in Northwest England, where 517,000 particles m^−2^ were observed on the River Tame (Hurley et al. 2018).

Concentrations in river sediments in non-European regions are generally higher compared to those observed in this study and are usually associated with urban and densely populated areas. For example, averages of 802 ± 59.4 MPs kg^−1^ were observed across seven urban rivers in Shanghai (Peng et al. 2018), with greater concentrations in densely populated areas compared to rural areas. In Changsha, concentrations ranged from 307.55 ± 94.73 to 580.79 ± 310.35 MPs kg^−1^ in urban waters across four tributaries to the Xiangjiang River that serves 7 million people with drinking water, although the relationship between MP abundances and distance to urban centres was not significant (Wen et al. 2018). Concentrations ranging from 833.33 ± 80.79 to 1633.34 ± 202.56 were observed in an urban river system in Central Mexico, with films and fragments comprising the bulk of pieces (Shruti et al. 2019).

**Fig. 7:**
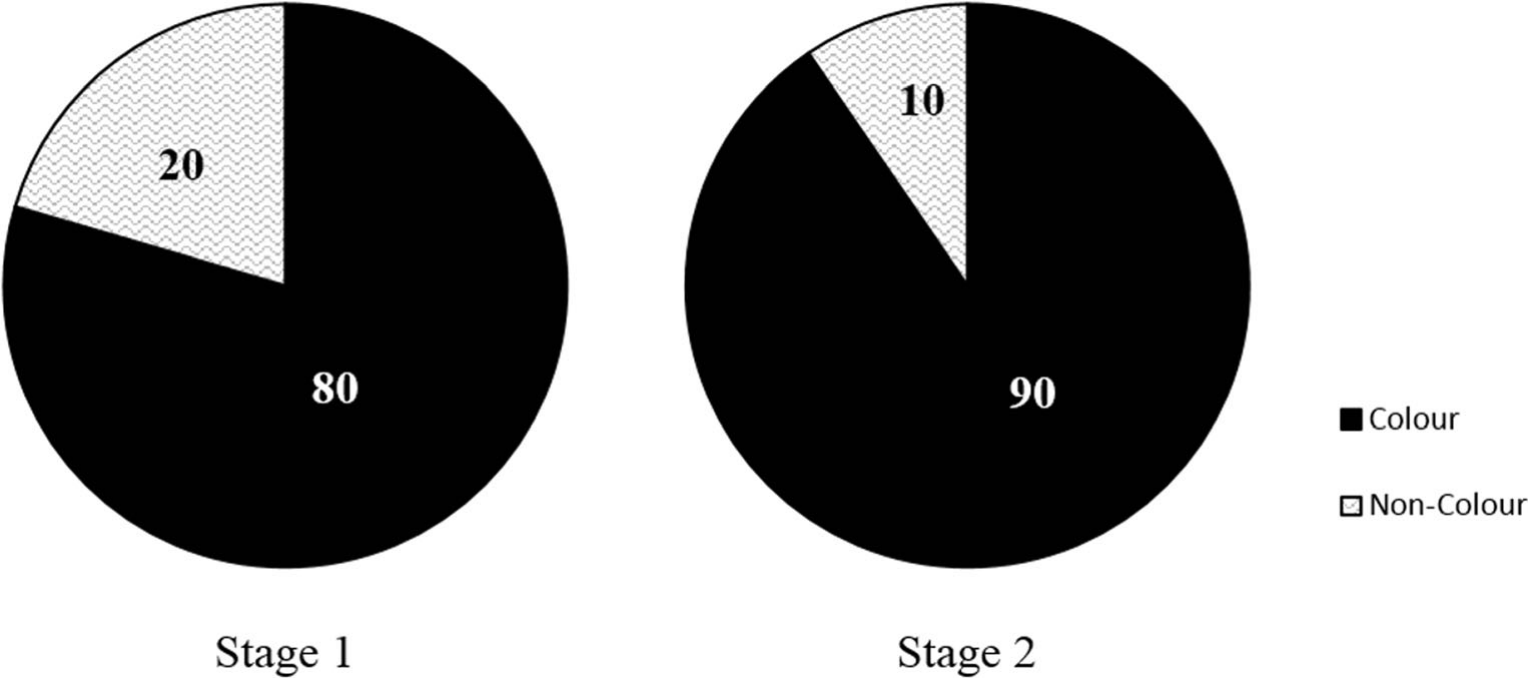
Percentages of coloured and non-coloured (i.e. white and translucent) pieces observed in River Kelvin sediment samples at each characterisation stage (data is pooled for both sampling events)

The relative abundance of secondary MP types observed here is also consistent with those from other freshwater studies conducted in Lake Hovsgol (Free et al. 2014), the Raritan River (Estahbanati and Fahrenfeld 2016) and urban Paris (Dris et al. 2015), although this comparison can only be expressed qualitatively, as different measurements and units were used. Methods and measurement units used in reporting results need harmonising for improved risk assessment and to facilitate discussion across studies. Nevertheless, the predominance of secondary MPs in the River Kelvin and other freshwater catchments supports the general assumption that most MPs in the environment originate from the breakdown of larger pieces (Duis and Coors 2016). Coloured pieces were more frequent than white and translucent pieces (Fig. 7), but further data is needed to determine whether this is an accurate reflection of their greater abundance in the environment, or if this is attributed to selection bias. Indeed, it has been suggested that fibre-like and bright-coloured pieces may be easier to find (Hidalgo-Ruz et al. 2012; Cole et al. 2014) and could be a source of analytical bias.

As the sampling site is a low-energy zone where sediment deposition tends to occur, the abundance of MPs here may support previous interpretations that processes affecting deposition of fine sediment similarly influence MPs (Vianello et al. 2013; Nizzetto et al. 2016) and may explain why fibres were more abundant and concentrated in the lower size fractions. Nevertheless, the distinctly different abundances observed between December and February samples in the River Kelvin suggest that high local variability can be expected, likely because MP contaminants encompass a wide array of highly diverse particles and thus will not be evenly distributed in space and time. The use of only one sampling site is a potential limitation of this study given the expected spatio-temporal variability of MPs in nature, and further spatial sampling and comparative data from the site and the local catchment are needed to improve our understanding of MP behaviour and distribution in this and similar freshwater systems. In addition, it is crucial to increase the spatial coverage of freshwater surveys through research like this, and the comparability across studies to fully understand this variability (Turra et al. 2014) and improve reliable assessment of their distribution and abundance in aquatic environments.

This research shows that freshwater river sediments close to marine estuary systems contain MPs, with fibres numerically dominant, and thus it is likely that freshwater systems are a feeder of marine MPs, mobilised for example to the marine environment by large flows (Nizzetto et al. 2016; Hurley et al. 2018). Moreover, the fate of MPs in these systems may be influenced by the association of different MP types and sizes with different sediment grain size fractions, and some MPs may be retained (Nizzetto et al. 2016). Thus, consideration of different particle-size fractions and areas where sediment accumulates is needed in river MP studies to improve understanding of MP emissions to oceans.

### Visual vs chemical characterisation

Counts and relative abundance of suspected MP types were used to compare the efficacy of visual and chemical characterisation techniques to discriminate plastics from other non-plastic microdebris and the sediment matrix before and after SEM-EDS analysis. Visually, identification of pieces that were different than sediment grains was possible by light microscopy although this was increasingly difficult in the fractions smaller than 0.125 mm due to decreasing resolution, and it was nearly impossible to distinguish plastic from non-plastic microdebris. As a result, visual characterisation may lead to overestimation of MP pieces due to misidentification, because floatation of non-polymer microdebris can occur and because non-plastic pellets and fragments can be easily confused for MP given their physical similarities. Visual inspection is often used in methodological approaches for initial enumeration and identification (Hidalgo-Ruz et al. 2012; Blair et al. 2017). However, heavy reliance on the visual and manual components at nearly every step of the process can introduce potential for selection bias (Cole et al. 2014) and is limited by what is reasonably visible with or without the aid of a microscope. While this detection limit will depend on the individual doing the identification, it is recommended that visual characterisation is not used for pieces smaller than 0.5 mm (Hidalgo-Ruz et al. 2012), a limit much higher than the lower limit set by sampling (e.g. 0.3 mm for neuston nets) and filtration (e.g. 0.7 μm for glass fibre filters) methods, including those used in this study.

Here, the chemical composition data from SEM-EDS was useful mainly for separation of non-plastic pellets and fragments in both suspended and settled material, but it was not useful for MP fibre identification. Further analysis by spectroscopy techniques such as Raman and FTIR-ATR (Blair et al. 2017) is likely necessary for proper MP fibre enumeration. While chemical characterisation by SEM-EDS and other complementary techniques like Raman and FTIR spectroscopy can aid to overcome detection limits and misidentification from visual characterisation (Wesch et al. 2016), it is important to note their limitations. First, these techniques can be extremely time-consuming and may be costly. For similar logistical reasons, it was possible only to analyse a microfibre sub-aliquot via SEM-EDS in this study. Care was taken to ensure that the sub-aliquot was representative of all types, colours, and size categories, but extrapolation of SEM-EDS results to the rest of the sample is undertaken visually and could result in some MP items being overlooked or misidentified. Second, chemical characterisation may be also subject to selection bias, as MP specimens needed to be isolated from other media and manually transferred to the instrument for analysis, depending on the ability of the researcher to first find these pieces visually. Lastly, instrument aided detection is also subject to size limitations. For Raman and FTIR, this is considered to be in the range of 0.5 and 10 μm, respectively (Hale 2017), although this may vary according to the equipment employed.

A combined approach that uses visual and multiple chemical characterisation techniques can address some of these methodological limitations. Combined or stepwise approaches are becoming more common in recent routine testing as a way to optimise extraction and characterisation methods and reduce analytical errors (Hidalgo-Ruz et al. 2012; Horton et al. 2017). Further, new studies are recognising the impact of visual reliance on size limitations and proper MP identification and are using advanced FTIR mapping techniques to develop automated methods (Primpke et al. 2017). This is an important step forward in method development because a lower size limit for MPs is yet to be established. In addition, automated methods will be crucial for emerging nanoplastic (< 100 nm) research that may become more abundant in the environment, as their use increases in future trends in technological applications and as macro- and microplastic waste continues to degrade (Koelmans et al. 2015).

## Conclusions

While MP pollution research is experiencing rapid development, research remains largely skewed towards marine systems with limited information for freshwater river compartments. As rivers receive anthropogenic waste inputs from the land they drain, they can act as important conduits of MPs from land-based sources to oceans and thus cannot be separated from marine MPs research. Therefore, this study contributes to a currently limited body of work exploring the concentration and composition of MPs in freshwater river sediment in close proximity to the marine environment. Furthermore, previous studies usually explore the correlation between MP concentrations and basin characteristics to identify potential sources, but this is one of the first to explore the associations of different types of MPs with different grain size fractions. This information contributes to understanding of the behaviour and fate of MPs in these systems to identify potential control points.

Results corroborate the ubiquity of MPs and suggest the predominance of secondary MPs, but high variability was observed in MP concentrations across sampling events during the same season. Fibres were always the dominant type of plastic, and while often associated with sewage discharge, their presence in this site suggests a greater contribution of other pathways, such as atmospheric deposition or in-stream transport. Nevertheless, this study focussed only on the exposed sediment fraction and a single sampling point, which are potential limitations; therefore, future work should expand on spatial sampling and incorporation of other environmental compartments to assess the extent of their spatio-temporal variability and the potential for storage vs transport of MPs in these systems. However, often, it may not be possible for researchers to include the samples needed for a comprehensive assessment of all liquid, solid, and gaseous fractions, thus research efforts should also aim to unify methodology for improved inter-comparison of available freshwater studies. Currently, methods can be subject to both under- and overestimation of different types of MPs, limiting comparability and potentially leading to inaccurate assessment of MP pollution, hindering risk assessment and possibly resulting in mitigation efforts that are largely misdirected. Further work is currently underway to examine the spatio-temporal distribution and chemical composition of MPs in a larger freshwater river system in the same catchment location reported in this paper. This study contributes to this further spatio-temporal survey by establishing a particle-size fraction profile of possible MPs in the catchment and refining the techniques needed to improve their extraction and identification.

## Electronic supplementary material

ESM 1 (DOCX 122 kb)
