# Supplementary material for: Microscopy and elemental analysis characterisation of microplastics in sediment of a freshwater urban river in Scotland, UK
Source: Environ Sci Pollut Res Int. 2019 Mar 8;26(12):12491–504. doi: 10.1007/s11356-019-04678-1 (PMC6476856; doi:10.1007/s11356-019-04678-1)
Supplement: Supplementary file 1 — (DOCX 122 kb) [file 11356_2019_4678_MOESM1_ESM.docx]

Electronic Supplementary Material

Article Title: Secondary microplastics were prevalent in sediment in a freshwater UK urban river

Authors: Reina M. Blair*, Susan Waldron, Vernon Phoenix, Caroline Gauchotte-Lindsay

*Corresponding author: School of Geographical and Earth Sciences

University of Glasgow, Glasgow G12 8QQ

email: r.blair.1@research.gla.ac.uk

**ESM 1** Visual (suspended and settled material) and chemical counts of suspected microplastics extracted from fractioned sediment samples collected from the River Kelvin on two sampling dates

**Visual counts, suspended**

**Visual counts, suspended (continued)**

**Visual counts, suspended (continued)**

**Visual counts, settled**

**Visual counts, settled (continued)**

**Visual counts, settled (continued)**

**Chemical counts, SEM-EDS**

**Chemical counts, SEM-EDS (continued)**

**Chemical counts, SEM-EDS (continued)**
